# Supplementary material for: Daily electric field treatment improves functional outcomes after thoracic contusion spinal cord injury in rats
Source: Nat Commun. 2025 Jun 26;16:5372. doi: 10.1038/s41467-025-60332-0 (PMC12202812; doi:10.1038/s41467-025-60332-0)
Supplement: Supplementary file 1 — Supplementary Information [file 41467_2025_60332_MOESM1_ESM.pdf]

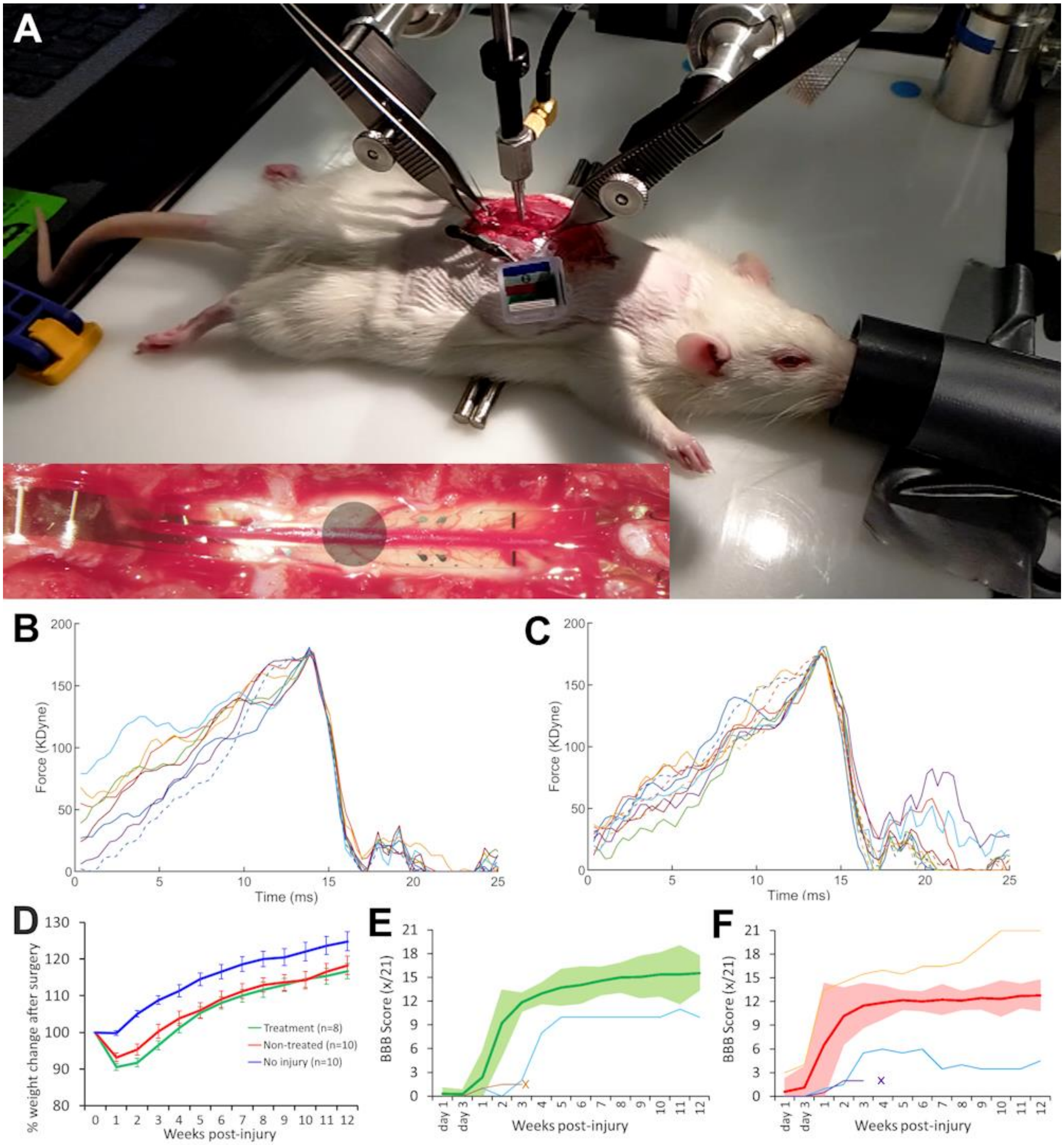
A
B
Force (KDyne)
Time (ms)
C
Force (KDyne)
Time (ms)
D
% weight change after surgery
Treatment (n=8)
Non-treated (n=10)
No injury (n=10)
Weeks post-injury
E
BBB Score (x/21)
Weeks post-injury
F
BBB Score (x/21)
Weeks post-injury

Supplementary Figure 1: Groups of treated and non-treated rats received a moderate 175 kilodyne impact injury to spinal segment L1/L2 using an Infinite Horizons Impactor. This moderate injury severity was chosen to allow treatment effects to be observed while ensuring mobility for tasks, avoiding long-term animal health issues, and aligning with the typical progression of viable treatments from moderate to severe injuries. (**A**) After subdural insertion of the implant, rats were placed on the impactor stage and held in position via hemostats clamped to the T9 and T13 spinal processes adjacent to the laminectomy. A 2.5 stainless steel impactor tip was used to deliver a 175 kilodyne impact to the boundary between spinal segments L1/L2 (directly below the T11 spinal process), which was centered between two sets of stimulation electrodes (shown in inset). Force vs time profiles of the impactions are shown for the (**B**) treated group (n = 8) and (**C**) non-treated group (n = 10). (**D**) In both the treated and non-treated groups rat's weight dropped immediately after surgery and then steadily increased; group means are shown +/- SEM. (**E**) Two rats in the treated group and (**F**) three rats in the non-treated group were excluded as their injury progression was two standard deviations outside the group mean; thick lines in **E, F** indicate group means with shading showing +/- SD; thin lines show individual rats; two implants from excluded rats were explanted intact at weeks 3 and 4 post-surgery and electrochemically tested (time of explantation indicated by crosses).

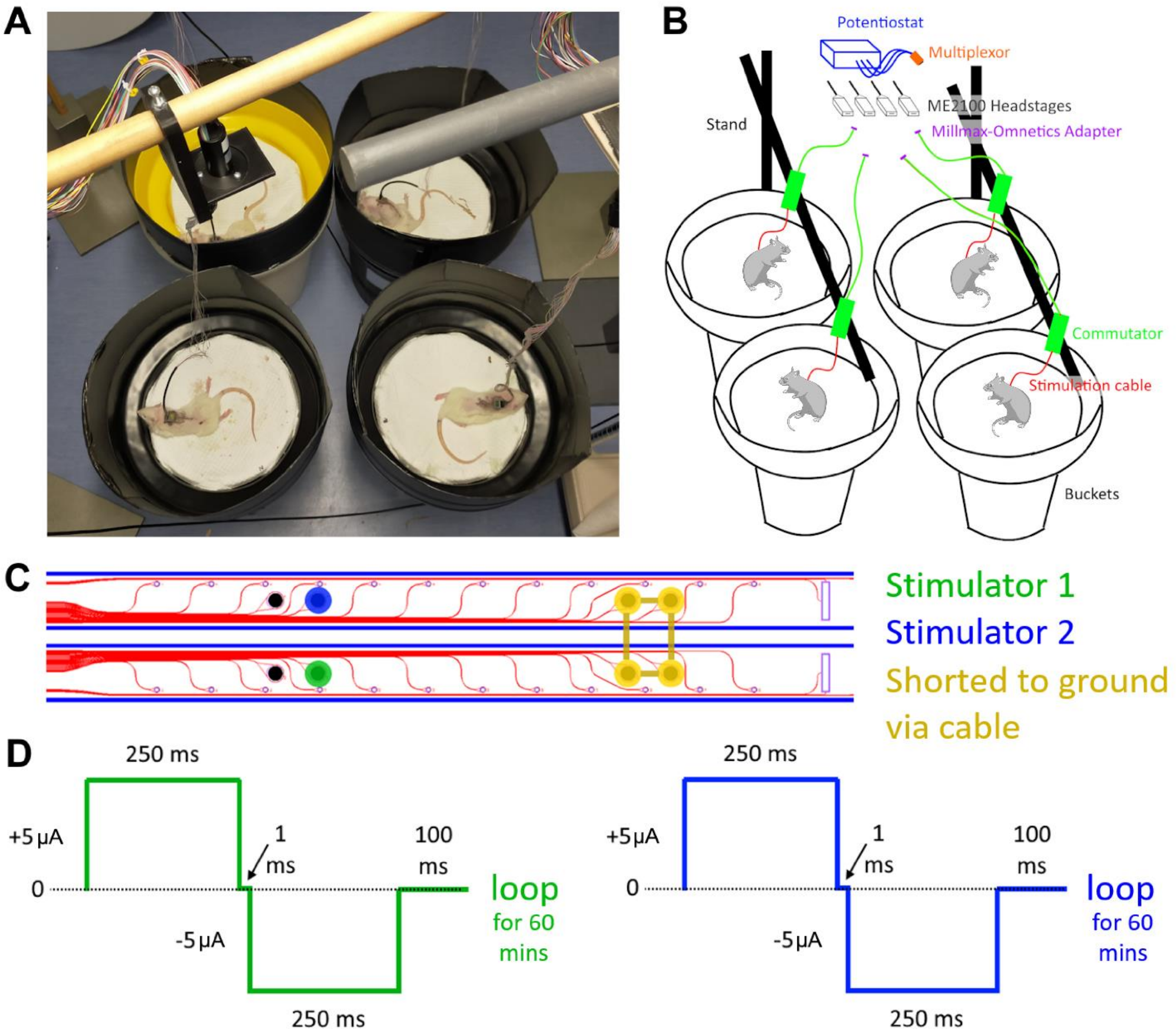

Supplementary Figure 2: Rats in the treated group received a daily one-hour EF treatment. (**A**) Up to four animals at a time were placed in comfortable towel-lined buckets and gently restrained while their implanted backpacks were connected to an overhanging stimulation cable. (**B**) The cables were connected to commutators, and each plugged into HS32 head stages and a ME2100 Multi Chanel Systems electrophysiology system for delivering stimulation. To test the impedance of electrodes, the cables exiting the commutators were instead plugged into a homemade multiplexor adapter and potentiostat. (**C**) Current-controlled ES was delivered via two stimulators in each headstage directed at stimulation electrodes on each arm of the implant (blue and green circles). The stimulation cables shorted the four electrodes on the caudal end to the ME2100 ground channel (yellow circles). (**D**) Each stimulator (green and blue) delivered a 1-hour electric field treatment consisting of looped ±5µA 250ms biphasic pulses with 100ms recharge periods across the injury site. Panel B contains images sourced from NIAID NIH BIOART Source. bioart.niaid.nih.gov/bioart/169

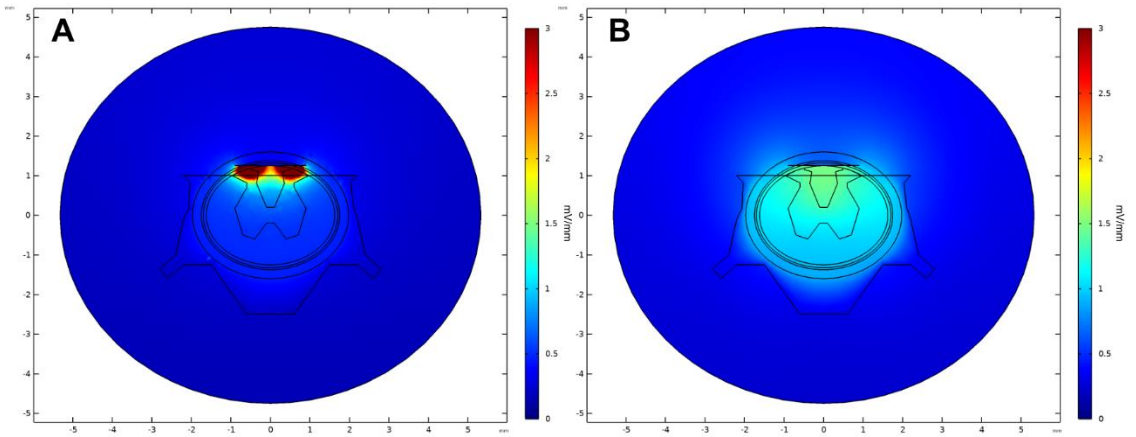

Supplementary Figure 3: Cross-sectional (coronal) perspective of FEM-based simulation of the medium sized rat's spinal cord. Longitudinal electric field strength is shown at (**A**) the level of the electrodes, and (**B**) equidistant between the electrodes at the center of the estimated spinal lesion**.** The electric field is more uniform and extends to the ventral outline of the cord at the midpoint between the electrodes, compared to its distribution directly at the electrode positions.

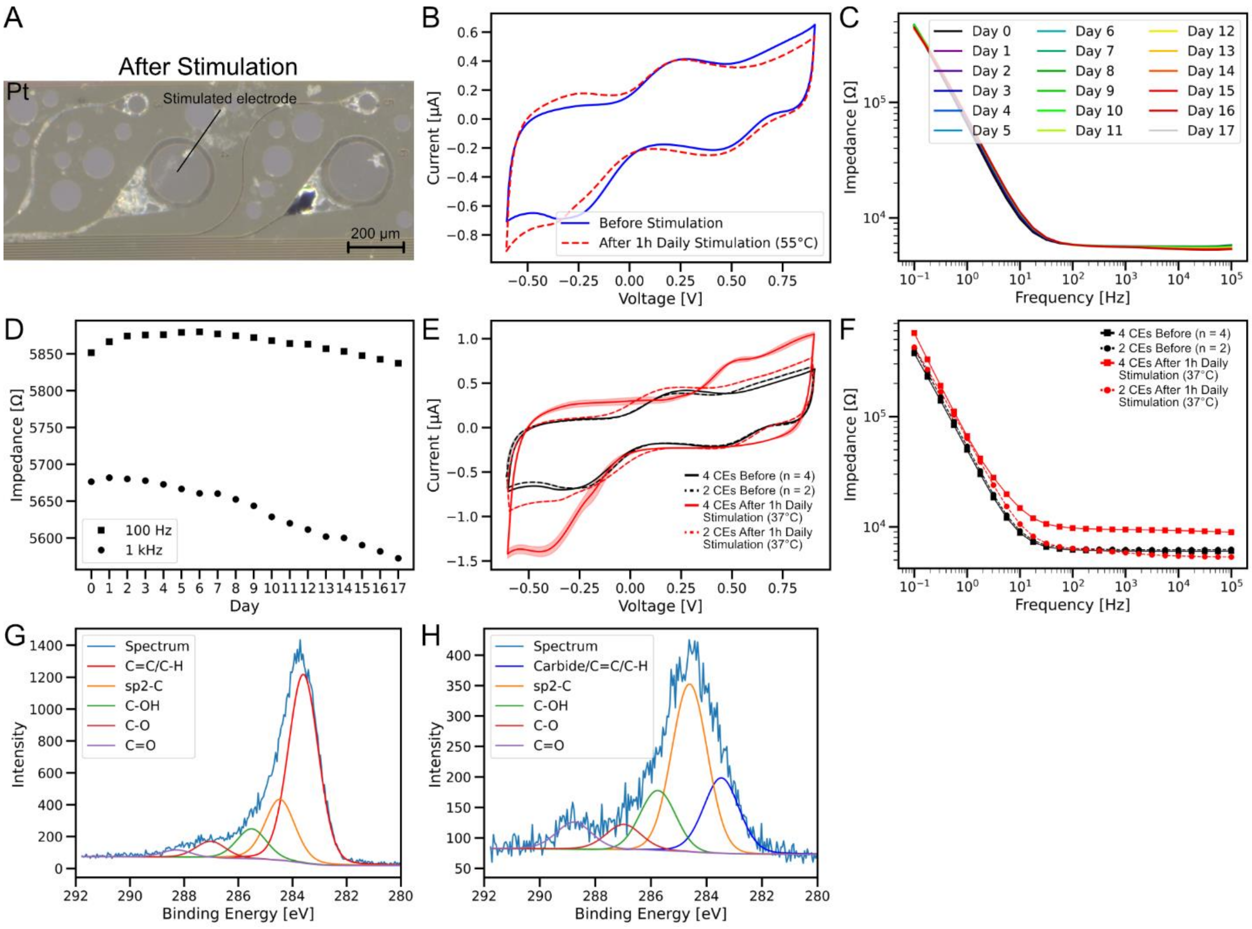

Supplementary Figure 4: The stability of Pt and SIROF stimulation electrodes was tested in 1x phosphate buffered saline using the same implant design but with fenestrations in the polyimide. (**A**) To test a Pt electrode, the SIROF coating was omitted. After 90 hours of continuous stimulation (5 µA, 250 ms, 2 Hz), the stimulated Pt electrode delaminated and dissolved, affecting neighboring recording and stimulation electrodes, which also delaminated and dissolved. We believe that over the course of the experiment first the stimulation electrodes dissolved. Then the Pt connection line was in contact with PBS and dissolved as well, causing a cross connection to neighboring channels. The *in vivo* treatment was simulated for 18 days at 55°C (equivalent to 62 days at 37°C according to the "10-degree rule") and for 60 days at 37°C. The same implant body was used for both temperatures, but different stimulation electrodes were tested. (**B, C, D**) After 18 days at 55°C with daily 1-hour stimulation, the SIROF electrode remained stable. (**E, F**) Although the four shorted SIROF counter electrodes used at both temperatures showed changes in their CV and EIS, they remained functional. On the two SIROF counter electrodes used to stimulate one working electrode, no layer deposited, and CV and EIS remained stable. (**G**) XPS spectrum of C1s peak revealed that carbon was electrodeposited in the long-term experiments. (**H**) XPS spectrum of C1s peak from non-stimulated electrode.

Supplementary Note 1: We used the WE from the second 60-day experiment for chemical analysis. Under an optical microscope, the deposited layer appeared similar to that observed in the previous experiment. However, the sample was not included in high-resolution imaging, as gold deposition would interfere with chemical analysis. The analysis revealed that the deposited layer primarily consists of C=C and/or C-H (**Supplementary Fig. 4G-H)**. The chemical composition of the deposited carbon is comparable to previous work where carbon was intentionally electrodeposited from a CO_2-methanol mixture under high pressure (>2 MPa) [68]. In that study, the proposed reaction was $CO_2 + 4H^+ + 4e^- \rightarrow C + 2H_2O$. Since no other carbon sources were present in our benchtop experiments, we hypothesize that a similar carbon reduction reaction occurred. Notably, iridium (Ir) has been reported as a catalyst for CO_2 reduction [69], supporting this hypothesis. In aqueous media, CO_2 reduction typically yields single-carbon compounds [70]. We thus hypothesize that the XPS C1s peak at 283.6 eV corresponds to C-H. The CO_2 reduction reaction in aqueous solutions competes with other reactions such as the hydrogen evolution reaction and the presumed IrOx reduction [71]. Additionally, CO_2 solubility in aqueous solutions is low, limiting the reaction rate [70]. High faradaic efficiency for CO_2 reduction is typically achieved in aprotic solvents or supercritical fluids [72]. Therefore, if we successfully activated and observed CO_2 reduction under these conditions, it is a remarkable finding.

Similar carbon deposition was not observed during 90h of continuous stimulation, indicating that the reaction is not driven by the stimulation but by the electrode potential after stimulation. In the CVs, carbon deposition is indicated by an increased reduction peak at -400 mV (**Fig. 5g, h**; **Supplementary Fig. 4E,F**) close to the -310 mV peak reported for carbon deposition from graphene oxide dispersions in PBS [73]. Indeed, this carbon reduction peak is in the range of the interpulse voltage (**Fig. 5c**). After stimulation, the electrode does not remain at that potential but gradually shifts toward the open-circuit potential over time [74]. However, it appears that the post-pulsing electrode potential falls within a narrow overpotential range that supports CO_2 reduction while preventing faster competing reactions that would otherwise depolarize the electrode more quickly, inhibiting CO_2 reduction. Although the exact deposition timeline is unknown, the slow accumulation of 300 nm over 60 days suggests a low deposition rate.

In summary, this low-voltage low-rate carbon electrodeposition on SIROF is an interesting phenomenon relevant to all studies involving SIROF stimulation. However, further investigation is beyond the scope of this work, as electrodes with carbon deposition remained functional, and we have not observed carbon deposition in electrodes used *in vivo* (**Supplementary Fig. 7**). Given the specific conditions required for CO_2 reduction, we find it unlikely that these conditions are replicated in CSF as they are in PBS. For example, while PBS uses a phosphate buffer, CSF is primarily buffered by HCO_3^- [32], affecting both proton availability and CO_2 solubility - two key factors influencing CO_2 reduction. We are keen to investigate this further and have started experiments along this line.

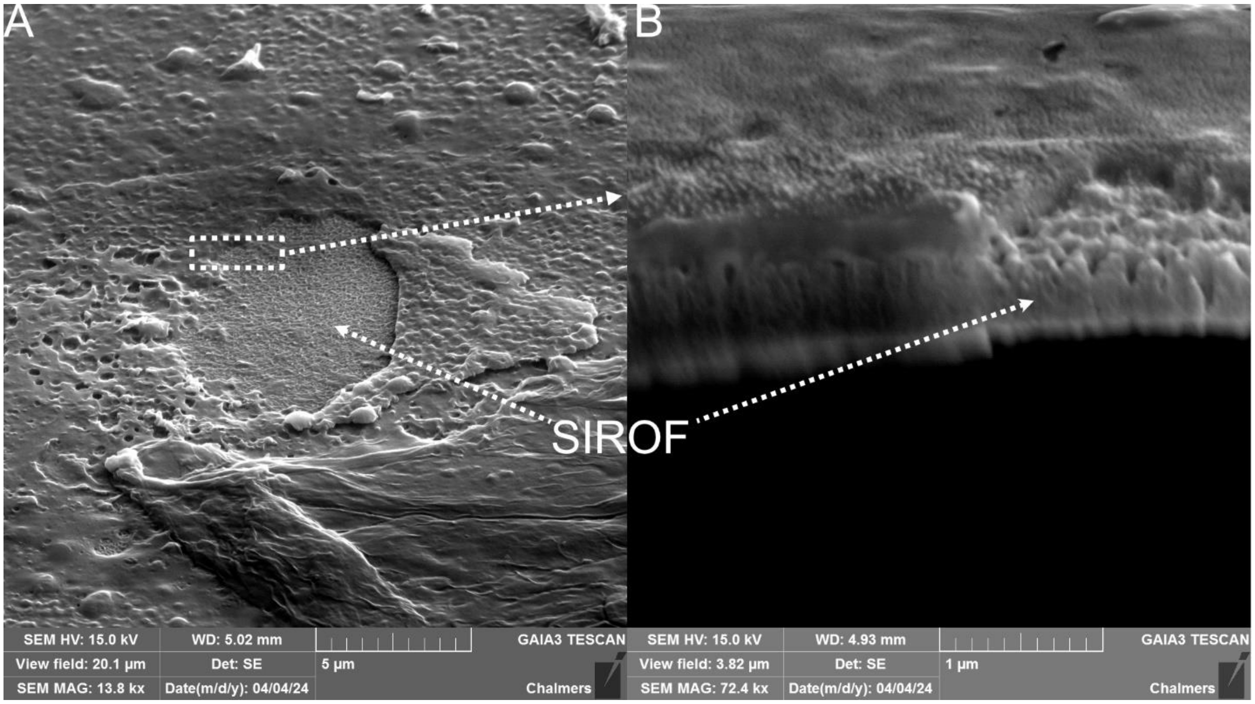

Supplementary Figure 5: CE SIROF electrodes exhibited surface densification and loss of fiber-like morphology during in vitro stimulation. (**A**) High resolution images of electrode surface. (**B**) FIB-SEM of the SIROF shows a deposited layer on top of the SIROF.

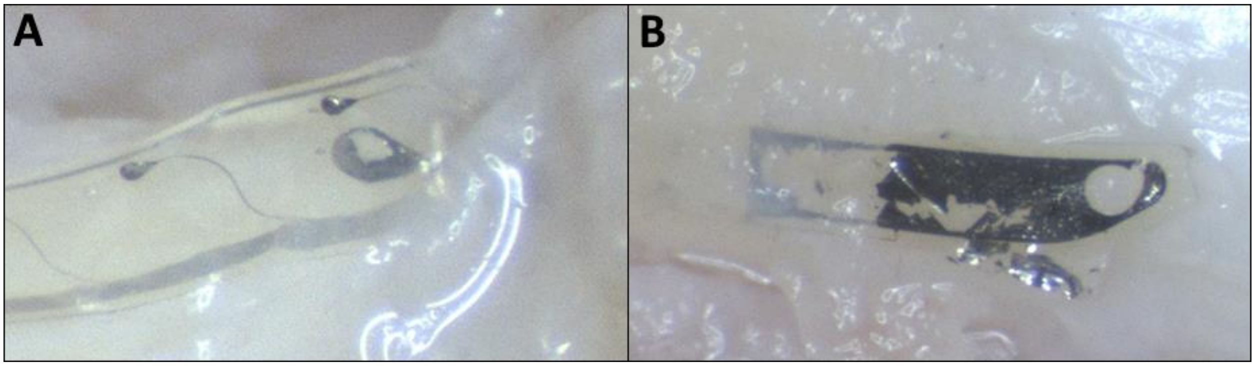

Supplementary Figure 6: During explantation of the device at 12 weeks, we observed delamination of larger electrode surfaces. This included the (**A**) stimulation electrodes and (**B**) large ground electrodes (used for recordings, not part of this study), which delaminated from the polyimide and/or stuck to the surface of the spinal cord. The stimulation electrode in A is subdural, the large electrode in B is outside the dura mater and spinal cord.

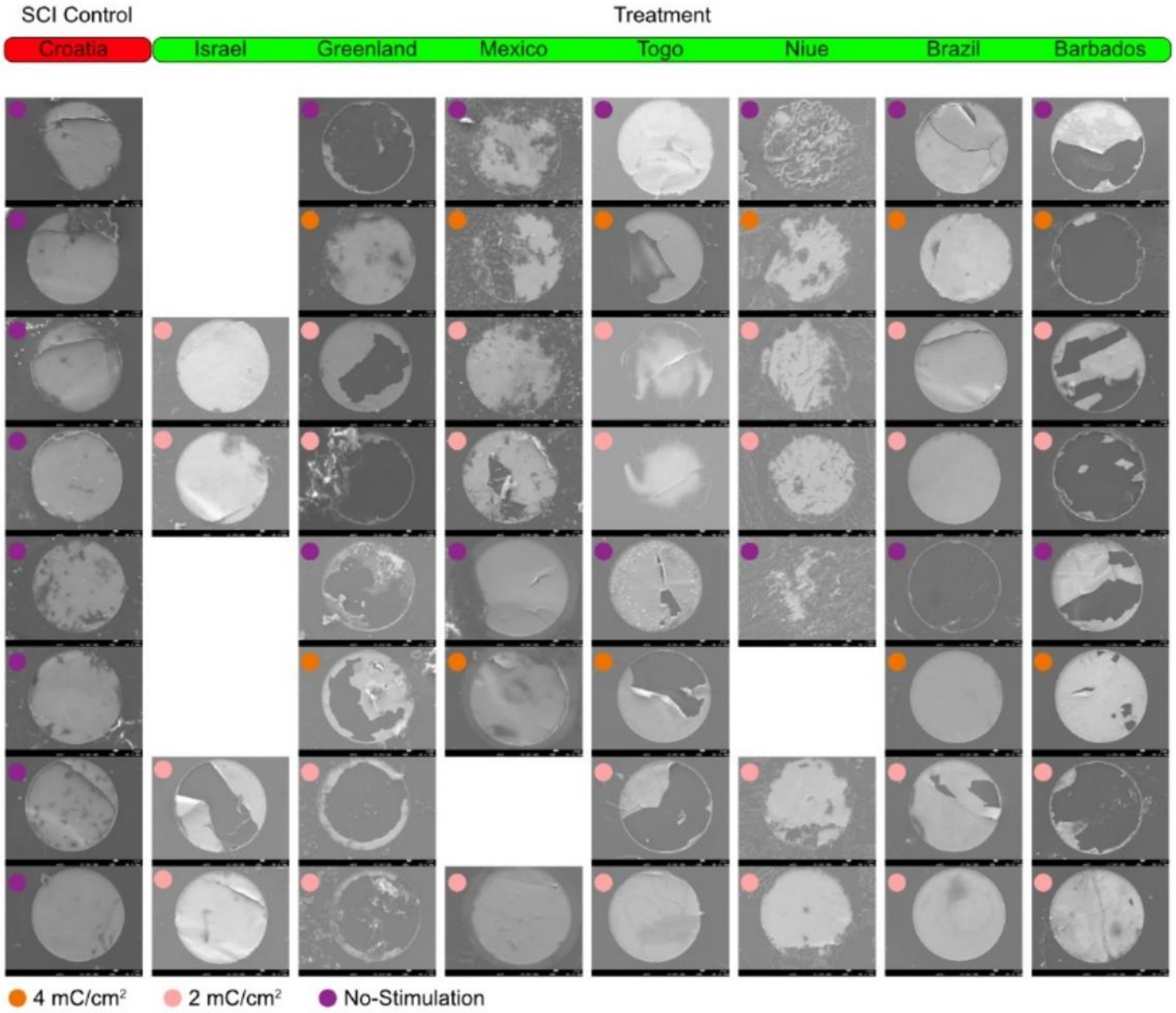

Supplementary Figure 7. SEM images of explanted devices.

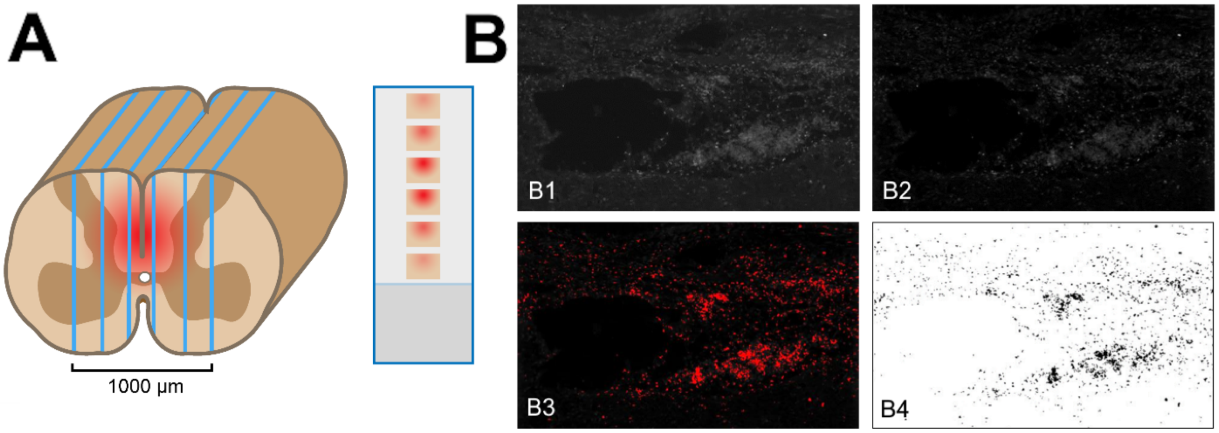

C

Antibody	Host	Target	Isotype	Supplier	Cat #	Dilution
**Anti-GFAP**	Mouse	Astrocytes (indicates lesion area)	IgG1	Sigma	G3893	1:500
**Anti-GFAP**	Rabbit	Astrocytes (indicates lesion area)	IgG	Sigma	G9269	1:500
**Anti- β-tubulin III**	Mouse	Neurons (indicating neuronal growth)	IgG2b	Sigma	T8660	1:500
**Anti-GAP43**	Rabbit	Neuronal growth	IgG	Abcam	ab16053	1:500
**Anti-5-HT**	Goat	Neuronal growth of Raphe spinal neurons	IgG	Abcam	ab66047	1:500
**Anti-IbA1**	Goat	Microglia (indicating neuroinflammation)	IgG	Abcam	ab5076	1:500

D

Antibody	Host	Target	Supplier	Cat #	Dilution	Excitation/ Emission
**Alexa Fluor™ 647**	Donkey	Anti-Mouse IgG	Thermo Fisher	A-31571	1:500	650/671 nm
**Alexa Fluor™ 594**	Donkey	Anti-Rabbit IgG	Thermo Fisher	A-21207	1:500	590/618 nm
**Alexa Fluor™ 488**	Donkey	Anti-Goat	Thermo Fisher	A-11055	1:500	499/520 nm

Supplementary Figure 8: Spinal cords from each group were sectioned and stained with antibodies to measure the degree of neuroinflammation and regeneration. (**A**) 20 µm thick sagittal sections were cut from 6 regions parallel to the midline of the 1 cm blocks of tissue and distributed onto slides. Image from BioDraws.com, released under a CC BY-SA 4.0 license. (**B**) Binary image of one channel/marker is shown in B1, background subtraction was performed in B2, threshold was applied in B3 to create a mask of fluorescence shown in B4. The area of fluorescently labeled tissue was then normalized against Hoechst 33342-positive cells to account for variations in tissue size. (**C**) The specific primary antibodies, their targets and dilutions are listed. (**D**) The three specific secondary antibodies that were used are listed, as well as their dilutions and excitation / emission rates. Panel A contains images from BioDraws.com

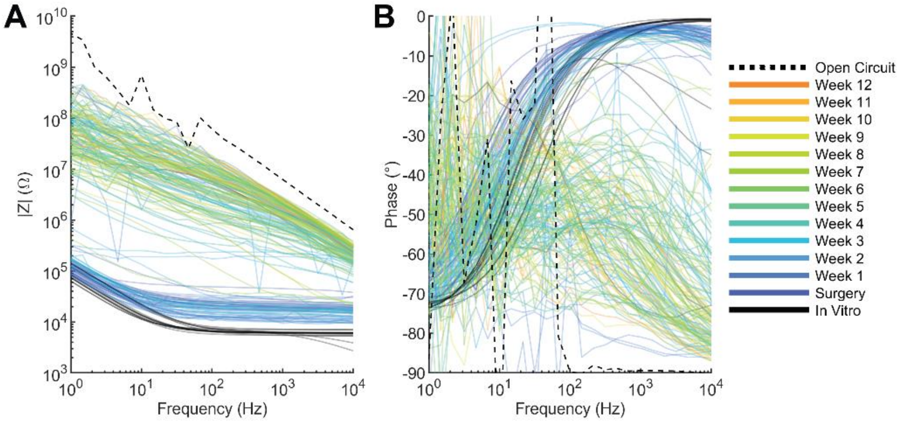

Supplementary Figure 9: Impedance of reserve electrodes that were not used for stimulation. (**A**) Impedance magnitude and (**B**) phase are shown over 12 weeks, indicated by the color scale, with the dashed red line representing the open circuit level.

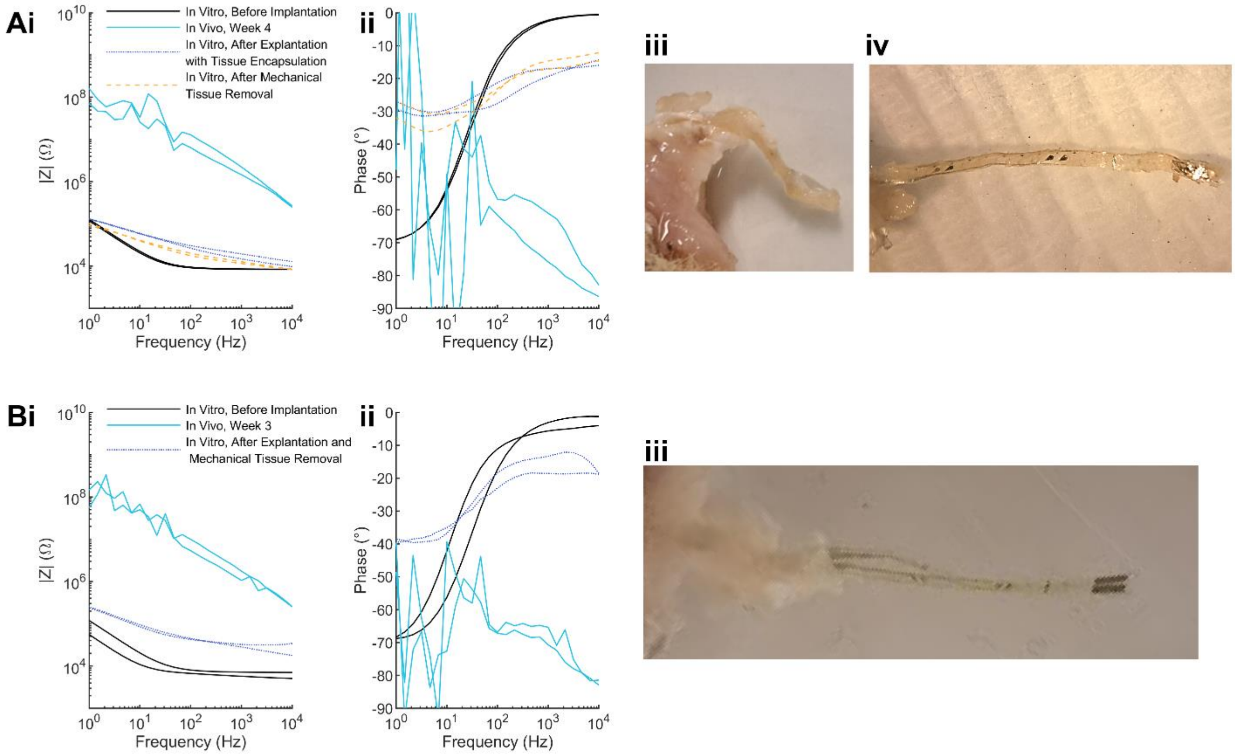

Supplementary Figure 10: Electrode impedance returned to lower levels after tissue removal in explants retrieved after 3-4 weeks. Explants were successfully recovered intact at **(A)** Week 4, and **(B)** Week 3. **(i-ii)** Impedance at various stages of examination. **(iii-iv)** Implants before and after removal of encapsulating tissue.

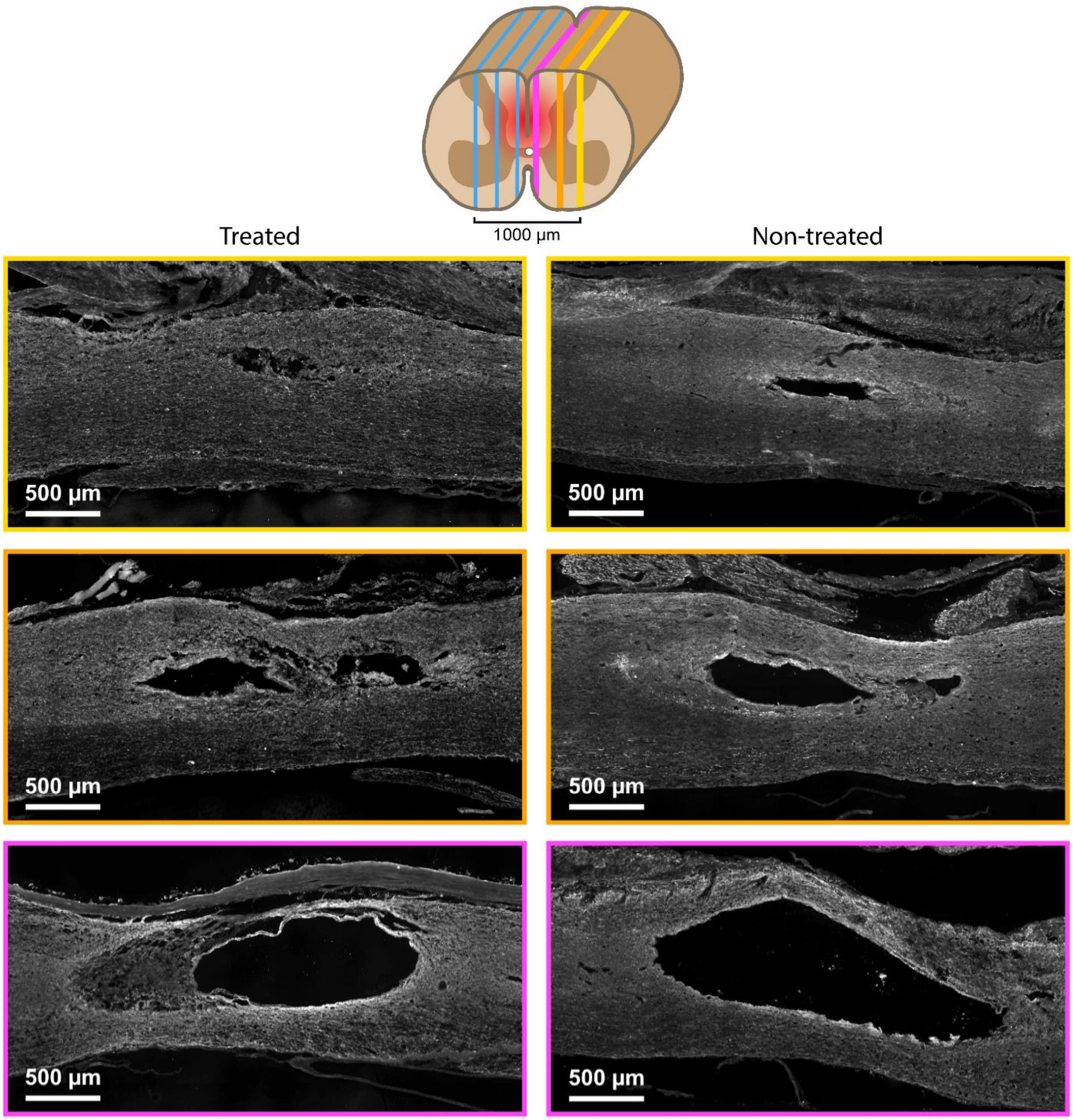

Supplementary Figure 11: Longitudinal sagittal sections of spinal cord showing representative cross-sections through the lesion in treated (left column) and non-treated (right column) rats. Panel A contains an image from BioDraws.com, released under a CC BY-SA 4.0 license.

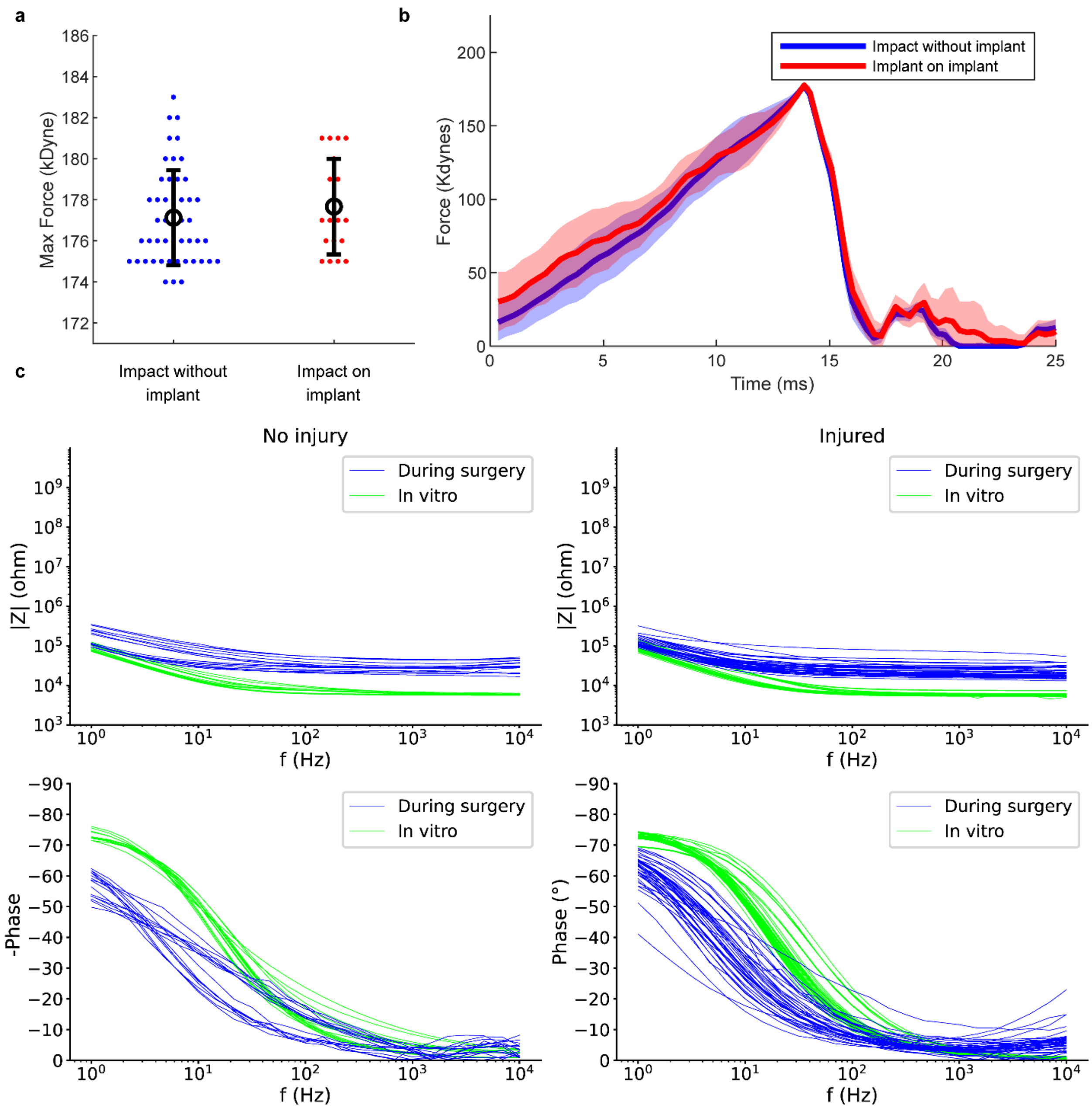

Supplementary Figure 12: Performing implant insertion followed by delivering the spinal cord impact directly on top of the implant within the same surgery does not alter the injury's impact mechanics or affect electrode impedance. This approach avoids potential damage from sliding the implant in after the impact when swelling and bleeding are present, and eliminates the need for multiple surgeries that could affect animal welfare and recovery. We compared implanted rats from this study (combined treated and non-treated groups) compared with animals from our previous study (Meissner et al., 2024) that underwent the same laminectomy and impact force / location, in the absence of an implant. (**A**) Comparison of max impact force (kDynes); mean +/- SD is shown, dots indicate individual rats. (**B**) Comparison of force over time mechanics of impact between rats with a subdurally positioned implant and those without an implant; mean +/- SD is shown. (**C**) Delivering the impact on top of the thin-film implant did not affect impedance measurements from the electrodes; we compare pre- and -post impaction impedance measurements from no-injury vs injury rats in the current study.

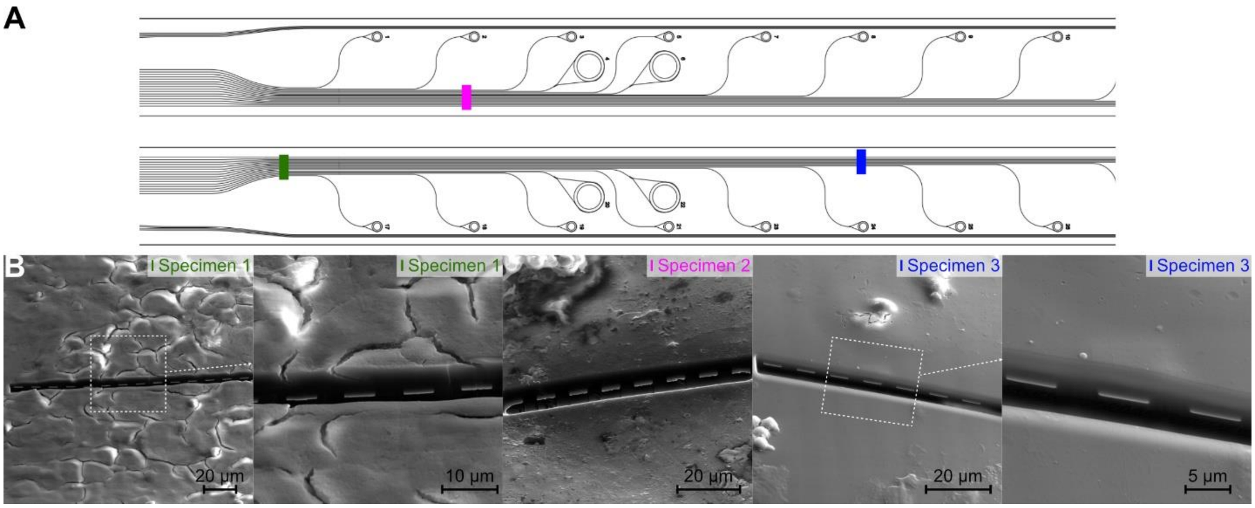

Supplementary Figure 13: High-resolution images of explants from the treated group showed that connection lines and polyimide insulation were intact. (**A**) Overview of the investigated regions on three randomly selected explants from the treated group. FIB cutting was targeted at PI areas with a high density of interconnection lines to enable simultaneous assessment of their status. (**B**) All examined interconnection lines remain intact.

References

68 Wu, M. *et al.* Electrochemical reduction of CO2 to carbon films on stainless steel around room temperature. *Electrochemistry Communications* **110**, 106606, doi:https://doi.org/10.1016/j.elecom.2019.106606 (2020).

69 Han, H., Jin, S., Park, S., Seo, M. H. & Kim, W. B. Atomic iridium species anchored on porous carbon network support: An outstanding electrocatalyst for CO2 conversion to CO. *Applied Catalysis B: Environmental* **292**, 120173, doi:https://doi.org/10.1016/j.apcatb.2021.120173 (2021).

70 Abbott, A. P. & Eardley, C. A. Electrochemical Reduction of CO2 in a Mixed Supercritical Fluid. *The Journal of Physical Chemistry B* **104**, 775-779, doi:10.1021/jp9932867 (2000).

71 Qiao, J., Liu, Y., & Zhang, J. (Eds.). *Electrochemical Reduction of Carbon Dioxide: Fundamentals and Technologies (1st ed.).* (CRC Press, 2016).

72 Melchaeva, O. *et al.* Electrochemical Reduction of Protic Supercritical CO(2) on Copper Electrodes. *ChemSusChem* **10**, 3660-3670, doi:10.1002/cssc.201701205 (2017).

73 Chen, L., Tang, Y., Wang, K., Liu, C. & Luo, S. Direct electrodeposition of reduced graphene oxide on glassy carbon electrode and its electrochemical application. *Electrochemistry Communications* **13**, 133-137, doi:https://doi.org/10.1016/j.elecom.2010.11.033 (2011).

74 Merrill, D. R., Bikson, M. & Jefferys, J. G. Electrical stimulation of excitable tissue: design of efficacious and safe protocols. *J Neurosci Methods* **141**, 171-198, doi:10.1016/j.jneumeth.2004.10.020 (2005).
