# Supplementary material for: Daily electric field treatment improves functional outcomes after thoracic contusion spinal cord injury in rats
Source: Nat Commun. 2025 Jun 26;16:5372. doi: 10.1038/s41467-025-60332-0 (PMC12202812; doi:10.1038/s41467-025-60332-0)
Supplement: Supplementary file 2 — Description of Additional Supplementary Files [file 41467_2025_60332_MOESM2_ESM.pdf]

Description of Additional Supplementary Files

File name: Supplementary Movie 1

Description: Representative locomotor behavior of treated and non-treated rats in the open field. The video presents examples from five treated rats (top panels, green borders) and five non-treated rats (bottom panels, red borders), each performing comparable runs. Each pair of runs is first shown at normal speed, followed by a replay at half speed to highlight gait and coordination details. All footage was recorded during the final two weeks of the study (week 11 or 12), illustrating locomotor performance at the end of the treatment period.
