# Supplementary material for: Daily electric field treatment improves functional outcomes after thoracic contusion spinal cord injury in rats
Source: Nat Commun. 2025 Jun 26;16:5372. doi: 10.1038/s41467-025-60332-0 (PMC12202812; doi:10.1038/s41467-025-60332-0)
Supplement: Supplementary file 4 — Reporting Summary [file 41467_2025_60332_MOESM4_ESM.pdf]

nature portfolio

Corresponding author(s): Bruce Harland

Last updated by author(s): Mar 17, 2025

Reporting Summary

Nature Portfolio wishes to improve the reproducibility of the work that we publish. This form provides structure for consistency and transparency in reporting. For further information on Nature Portfolio policies, see our Editorial Policies and the Editorial Policy Checklist.

Statistics

For all statistical analyses, confirm that the following items are present in the figure legend, table legend, main text, or Methods section.

n/a	Confirmed	
☐	☒	The exact sample size (*n*) for each experimental group/condition, given as a discrete number and unit of measurement
☐	☒	A statement on whether measurements were taken from distinct samples or whether the same sample was measured repeatedly
☐	☒	The statistical test(s) used AND whether they are one- or two-sided<br>*Only common tests should be described solely by name; describe more complex techniques in the Methods section.*
☒	☐	A description of all covariates tested
☒	☐	A description of any assumptions or corrections, such as tests of normality and adjustment for multiple comparisons
☐	☒	A full description of the statistical parameters including central tendency (e.g. means) or other basic estimates (e.g. regression coefficient) AND variation (e.g. standard deviation) or associated estimates of uncertainty (e.g. confidence intervals)
☐	☒	For null hypothesis testing, the test statistic (e.g. *F*, *t*, *r*) with confidence intervals, effect sizes, degrees of freedom and *P* value noted<br>*Give P values as exact values whenever suitable.*
☒	☐	For Bayesian analysis, information on the choice of priors and Markov chain Monte Carlo settings
☒	☐	For hierarchical and complex designs, identification of the appropriate level for tests and full reporting of outcomes
☒	☐	Estimates of effect sizes (e.g. Cohen's *d*, Pearson's *r*), indicating how they were calculated

Our web collection on statistics for biologists contains articles on many of the points above.

Software and code

Policy information about availability of computer code

Data collection	IH Spinal Cord Impactor v5 (Precision Systems and Instrumentation) was used to deliver impact injury and record impact mechanics. Multi Channel Systems Experimenter Software was used to apply stimulation. Nova 2.1.6 (Metrohm Autolab) was used to record impedance data. MetaFer V3.12.1 (MetaSystems) was used to capture histology images on a VSlide Scanner. COMSOL Multiphysics 5.3 was used for finite element modelling of the implant and rat spinal cord.
Data analysis	Microsoft Excel (2502), Graphpad Prism 10, and Matlab R2022B were used for data analysis

For manuscripts utilizing custom algorithms or software that are central to the research but not yet described in published literature, software must be made available to editors and reviewers. We strongly encourage code deposition in a community repository (e.g. GitHub). See the Nature Portfolio guidelines for submitting code & software for further information.

Data

Policy information about availability of data

All manuscripts must include a data availability statement. This statement should provide the following information, where applicable:

- Accession codes, unique identifiers, or web links for publicly available datasets
- A description of any restrictions on data availability
- For clinical datasets or third party data, please ensure that the statement adheres to our policy

The data and code used to generate the main and supplementary figures are provided in the source data file.

Research involving human participants, their data, or biological material

Policy information about studies with human participants or human data. See also policy information about sex, gender (identity/presentation), and sexual orientation and race, ethnicity and racism.

Reporting on sex and gender	NA
Reporting on race, ethnicity, or other socially relevant groupings	NA
Population characteristics	NA
Recruitment	NA
Ethics oversight	NA

Note that full information on the approval of the study protocol must also be provided in the manuscript.

Field-specific reporting

Please select the one below that is the best fit for your research. If you are not sure, read the appropriate sections before making your selection.

☒ Life sciences ☐ Behavioural & social sciences ☐ Ecological, evolutionary & environmental sciences

For a reference copy of the document with all sections, see nature.com/documents/nr-reporting-summary-flat.pdf

Life sciences study design

All studies must disclose on these points even when the disclosure is negative.

Sample size	Sample size was determined using a power analysis based on expected effect sizes from prior studies. A power level of 80% and desired alpha level of 0.02 were used to estimate the required number of animals per group. Calculations were performed using the sample size spreadsheet on the Boston University Research Support Website.
Data exclusions	Five subjects were identified as statistical outliers and were removed from the study on the basis of post-surgical BBB scores that varied more than two standard deviations from their group mean (treated group, n = 2; non-treated group, n = 3); see Supplementary Fig. 1E-F. Two of the outlier rats were perfused in week 3 and 4 to examine electrode impedance of the explanted device
Replication	Animals were assigned to experimental groups across multiple cohorts, the treatment was not repeated as a separate independent experiment. Data were internally consistent across subjects.
Randomization	All animals had identical baselines as they were uninjured. Post-injury, treatment was assigned randomly based on surgery order, which was loosely weight-based (heavier-to-lighter) to ensure comparable weights across several weeks of surgeries.
Blinding	BBB and error ladder assessments were performed on videos where DeepLabCut blinded the identity of individual animals. Von Frey withdrawal thresholds were determined automatically by the apparatus, minimizing experimenter bias.

Reporting for specific materials, systems and methods

We require information from authors about some types of materials, experimental systems and methods used in many studies. Here, indicate whether each material, system or method listed is relevant to your study. If you are not sure if a list item applies to your research, read the appropriate section before selecting a response.

Materials & experimental systems

n/a	Involved in the study	
☐	☒	Antibodies
☒	☐	Eukaryotic cell lines
☒	☐	Palaeontology and archaeology
☐	☒	Animals and other organisms
☒	☐	Clinical data
☒	☐	Dual use research of concern
☒	☐	Plants

Methods

n/a	Involved in the study	
☒	☐	ChIP-seq
☒	☐	Flow cytometry
☒	☐	MRI-based neuroimaging

Antibodies

Antibodies used	Anti-GFAP (mouse, Sigma, G3893), Anti-GFAP (rabbit, Sigma, G9269), Anti- B-tubulin III (mouse, Sigma, T8660), Anti-GAP43 (rabbit, Abcam, ab16053), Anti-5-HT (goat, Abcam, ab66047), Anti-IbA1 (goat, Abcam, ab5076), Alex Fluor 647 (donkey, Thermo Fisher, A-31571), Alexa Fluor 594 (donkey, Thermo Fisher, A-21207), Alexa Fluor 488 (donkey, Thermo Fisher, A-11055)
Validation	Primary antibodies used in this study (from Sigma and Abcam) were validated by the manufacturer for the specified species and application. Additionally, all antibodies were tested in our lab for specificity and performance prior to use in this study.

Animals and other research organisms

Policy information about studies involving animals; ARRIVE guidelines recommended for reporting animal research, and Sex and Gender in Research

Laboratory animals	2 – 3 month old female Sprague Dawley rats
Wild animals	NA
Reporting on sex	These initial experiments of subdural EF treatment were performed in female rats only. Our primary concern with using male rats is the challenge of managing bladder function post-surgery. Other researchers locally and in other labs have experienced significant issues with infections and loss of animals following spinal cord injury in male rats. An additional issue is increased tendency for weight gain in males, which tends to disproportionately increase rear body mass relative to the front, which can negatively impact their ability to recover hind-limb function. Furthermore, the variability in weight gain among individual males introduces another layer of complexity, potentially affecting the interpretation of hind-limb recovery outcomes. This challenge is amplified by the long 12-week experimental timeline required to assess the efficacy of EF treatments. While food restriction could mitigate some of these issues, we prefer to avoid this approach at this stage of our research. However, we acknowledge the importance of demonstrating efficacy in males, particularly due to the higher prevalence of SCI in this population and intend to address this in future work.
Field-collected samples	NA
Ethics oversight	the University of Auckland Animal Ethics Committee, this work was approved by them (AEC22644) under the guidelines of the New Zealand Animal Welfare Act 1999.

Note that full information on the approval of the study protocol must also be provided in the manuscript.

Plants

Seed stocks	NA
Novel plant genotypes	NA
Authentication	NA
