# Supplementary figures and images for: Daily electric field treatment improves functional outcomes after thoracic contusion spinal cord injury in rats
Source: Nat Commun. 2025 Jun 26;16:5372. doi: 10.1038/s41467-025-60332-0 (PMC12202812; doi:10.1038/s41467-025-60332-0)

No injury

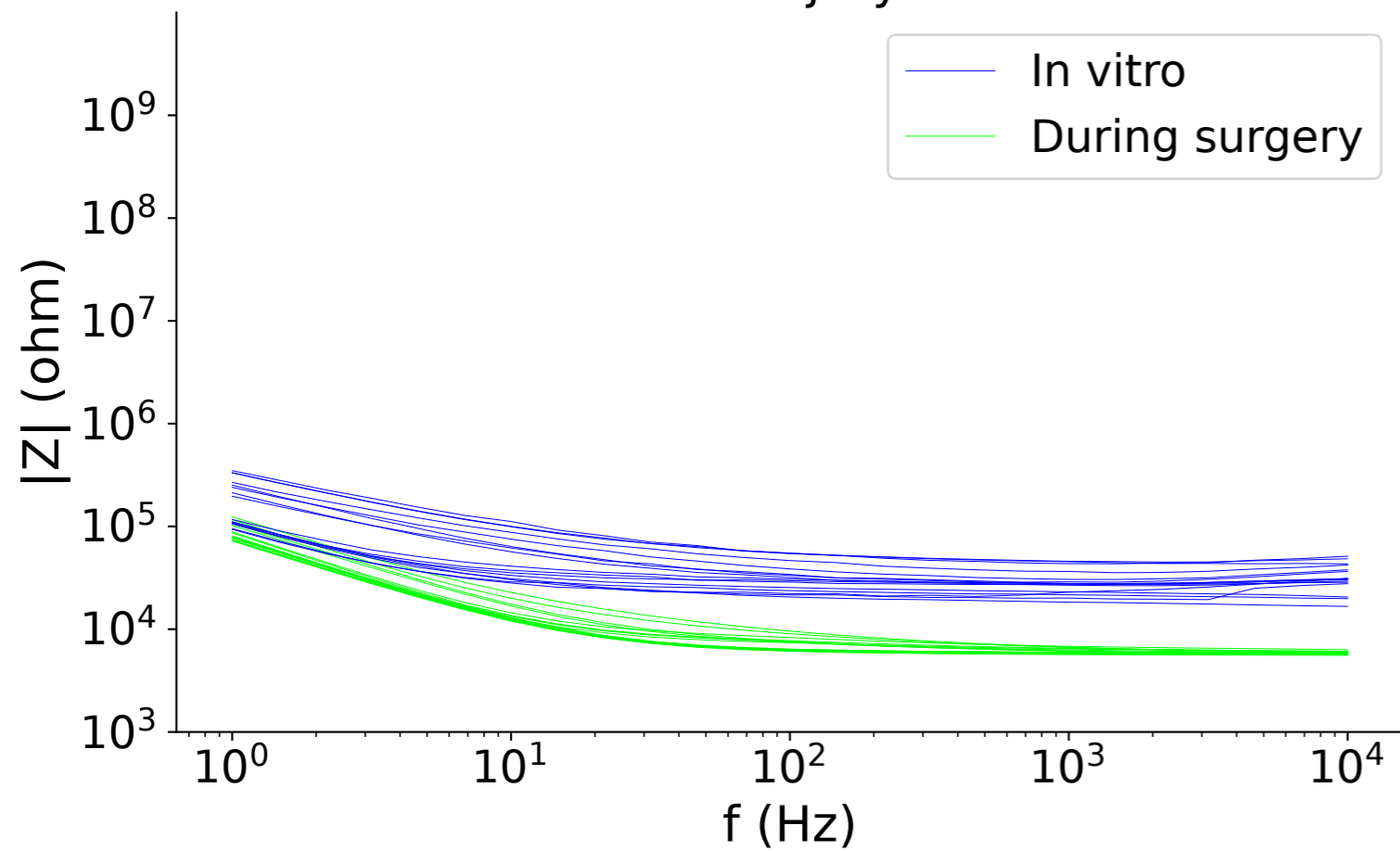

Injured

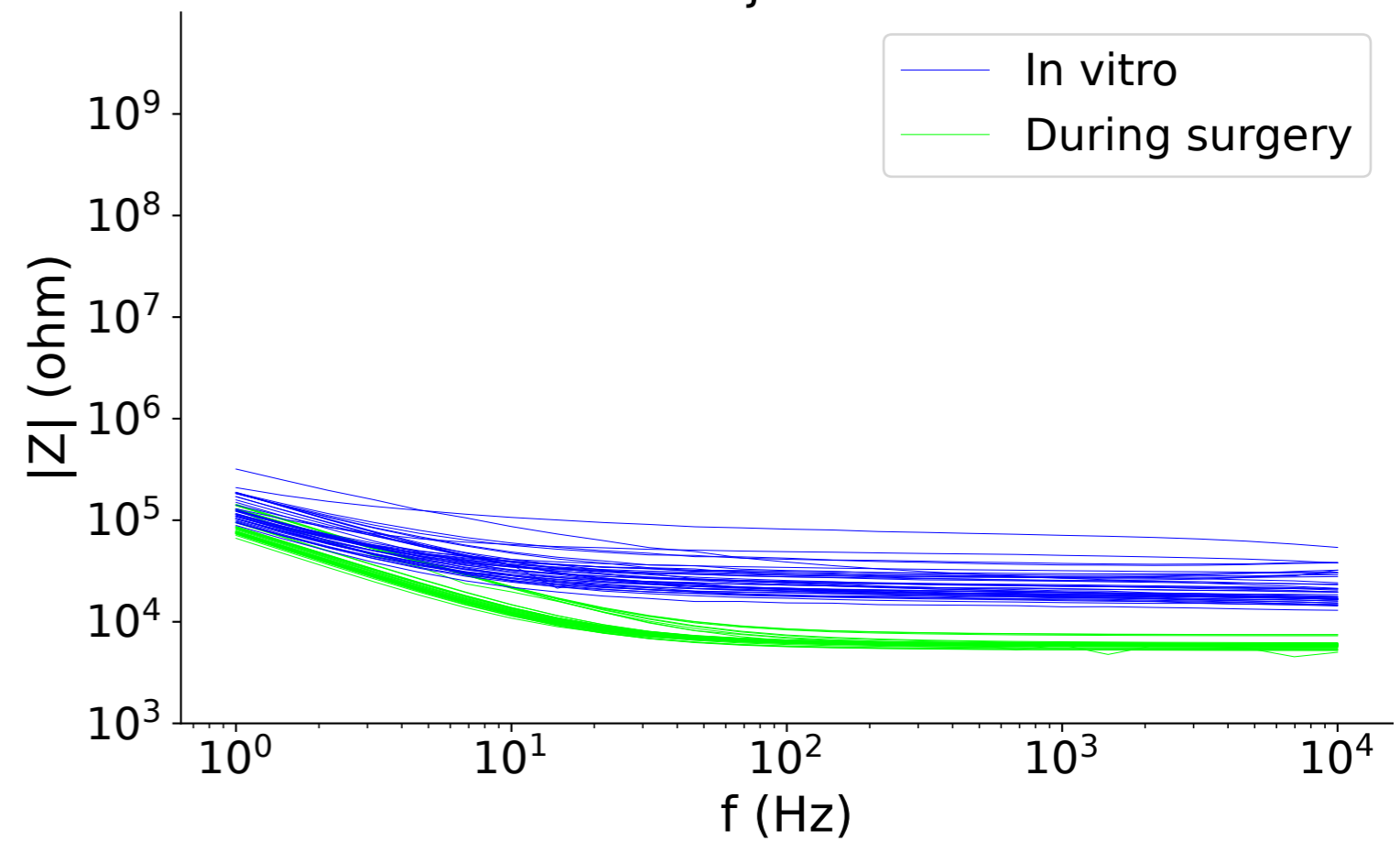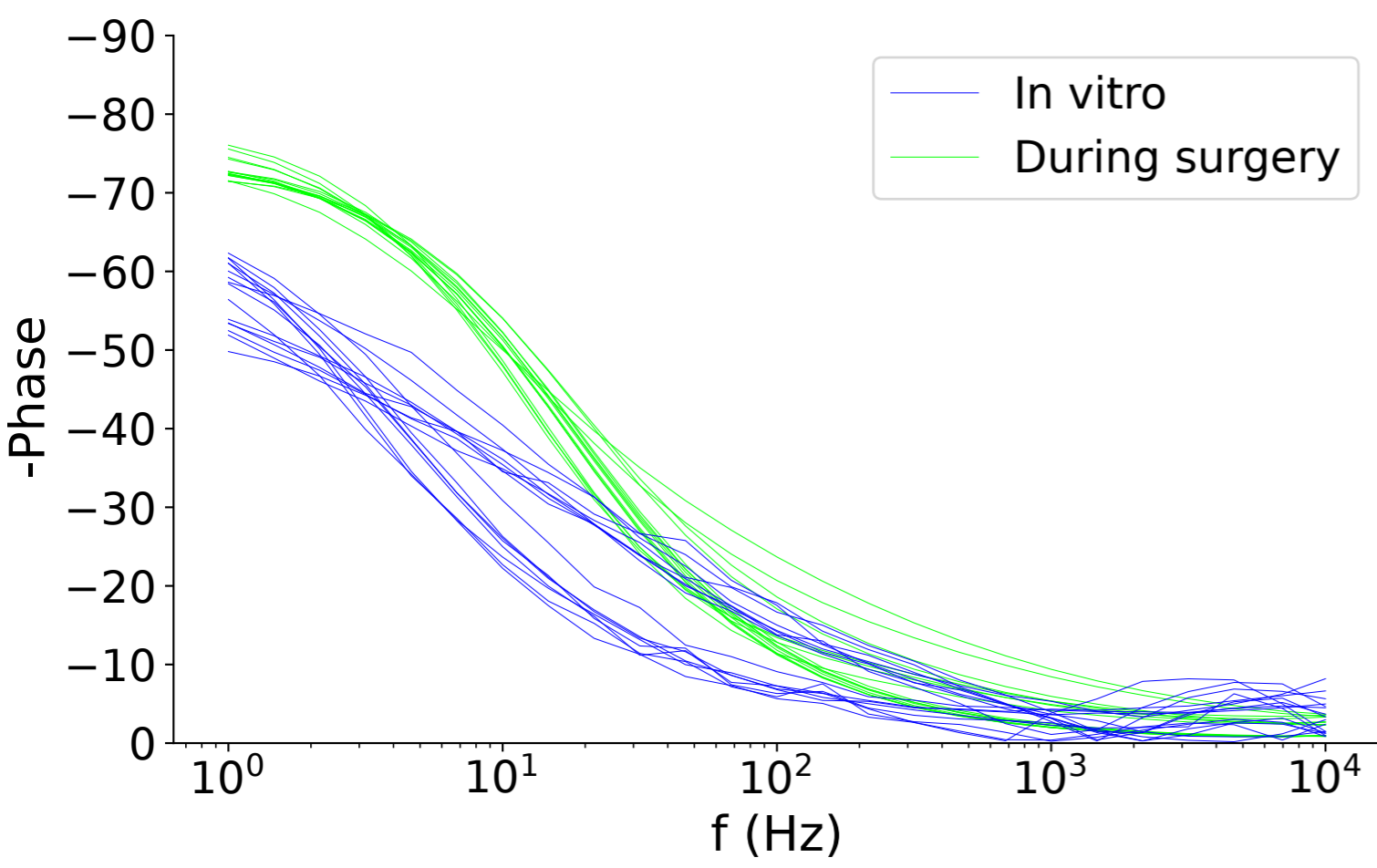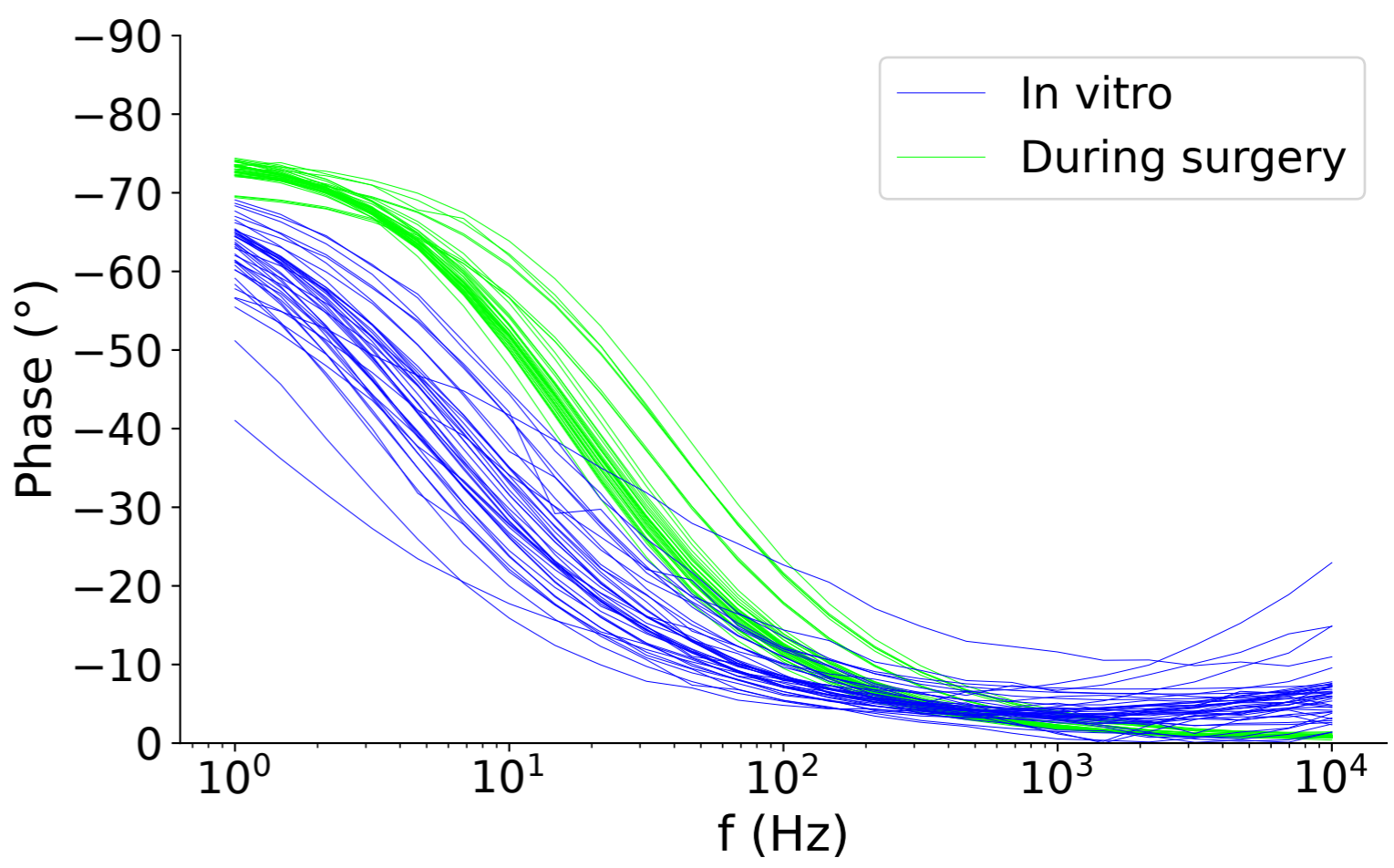

Supplement: Supplementary file 6 — Source Data [file 41467_2025_60332_MOESM6_ESM.zip › SFig12C.pdf]
